# Appetitive and aversive social learning with living and dead conspecifics in crickets

**DOI:** 10.1038/s41598-020-66399-7

**Published:** 2020-06-09

**Authors:** Hiroki Ebina, Makoto Mizunami

**Affiliations:** 1 Graduate School of Life Science, Hokkaido University, Sapporo, 060-0810 Japan; 2 Faculty of Science, Hokkaido University, Sapporo, 060-0810 Japan

**Keywords:** Neuroscience, Learning and memory, Social behaviour

## Abstract

Many animals acquire biologically important information from conspecifics. Social learning has been demonstrated in many animals, but there are few experimental paradigms that are suitable for detailed analysis of its associative processes. We established procedures for appetitive and aversive social learning with living and dead conspecifics in well-controlled stimulus arrangements in crickets, *Gryllus bimaculatus*. A thirsty demonstrator cricket was released in a demonstrator room and allowed to visit two drinking apparatuses that contained water or saltwater and emitted apple or banana odour, and a thirsty learner was allowed to observe the demonstrator room through a net. In the post-training test, the learner preferred the odour of the water-containing apparatus at which the demonstrator stayed. When a dead cricket was placed on one of the two apparatuses, the learner avoided the odour of that apparatus. Further experiments suggested that a living conspecific can be recognized by either visual or olfactory cues for appetitive social learning, whereas olfactory cues are needed to recognize a dead conspecific for aversive social learning, and that different associative processes underlie social learning with living and dead conspecifics. The experimental paradigms described here will pave the way for detailed research on the neural basis of social learning.

## Introduction

Social learning, in which animals learn from the behaviour of other animals, has advantages for the survival of animals since it helps them to find food and mates as well as to avoid predators or other forms of danger. Social learning has been found in many animals including insects^1–4^. Well-established examples of social learning paradigms in insects include bumblebees finding profitable flowers^2,5,6^ or avoiding flowers that harboured predators by observing a nestmate’s flower choice^7^ and mate-choice copying by female fruit flies^8^, which contributed to extend our knowledge on adaptive significance of social learning. However, there are few experimental paradigms suitable for detailed analysis of the nature of associative processes that underlies social learning in animals. As a result, studies on neural mechanisms of social learning in animals have only recently started to be conducted^9–11^.

The capability to discriminate living and dead conspecifics is also a fundamental capability of animals including insects^12–14^. In many aggregative insects, different chemical cues are used for the recognition of living and dead conspecifics, and signals for the former allow communications among conspecifics, whereas signals for the latter serve for avoiding disease, predation or poisonous food^12,13^. Thus, it is most likely that many animals can learn differently from living and dead conspecifics. However, to our knowledge, experimental paradigms that allow detailed investigation of such capability for achieving social learning have not been established in any animals.

In this study we investigated the capability of the cricket *Gryllus bimaculatus*, a subsocial insect that lives relatively solitary but exhibits sophisticated communications among conspecifics^15^, to learn from a living or dead conspecific. Crickets have emerged as useful model animals for the study of individual (asocial) learning since they have excellent capability of Pavlovian conditioning^16,17^ and since application of experimental manipulations, such as pharmacology, RNAi and genome editing, is feasible^18–23^. For example, we have shown that octopamine and dopamine neurons mediate appetitive and aversive reinforcing signals in Pavlovian conditioning of odour, visual pattern or colours (conditioned stimulus, CS) with water or saltwater (appetitive or aversive unconditioned stimulus, US)^18–21^. Here we developed procedures of social learning paradigms by modifying procedures for Pavlovian conditioning to associate an odour CS with water US or saltwater US to lead to increased or decreased preference for the odour, respectively^16,18,19^, in order to facilitate comparison of associative processes underlying social learning with those underlying Pavlovian conditioning in crickets.

In one experiment, a thirsty cricket (demonstrator) was allowed to freely visit two drinking apparatuses that contained water or saltwater and emitted apple or banana odour (counterbalanced). Another thirsty cricket (learner) was allowed to observe the demonstrator’s room through a net. The learners were tested for their relative preference between the two odours at 24 hours after training. The basic rationale of the experiment was that crickets, like other insects, are capable of perceiving a nearby odour source by sensing the odour by olfactory receptors on their antennae and are also capable of detecting a nearby water source by sensing water vapour by antennal hygroreceptors^24,25^. However, they are not able to discriminate water from saltwater since NaCl does not evaporate. Thus, our experiment was designed to examine, in a subsequent test, whether a learner that observed a demonstrator staying at a water source would show a higher preference for the odour of the apparatus at which the demonstrator stayed, which indicates social learning. Strictly speaking, the vapor pressure of the NaCl solution is slightly lower than that of water and this might inform crickets that the amount of water is smaller in the NaCl solution. However, we did not observe that the learners learn to select the odour of water-containing apparatus over the odour of NaCl solution-containing apparatus when a demonstrator is absent (see Results section). In a second series of experiments, a freshly sacrificed cricket was placed in one of the two drinking apparatuses that emitted apple or banana odour and a learner was allowed to observe the demonstrator room. We found that crickets achieve appetitive social learning with a living conspecific and aversive social learning with a dead conspecific. We further investigated sensory cues necessary to recognize a living or dead conspecific and possible associative processes underlying appetitive or aversive social learning in crickets, which will provide a basis for future studies on neural mechanisms of social learning.

## Results

### Social learning with a living conspecific

**Figure 1:**
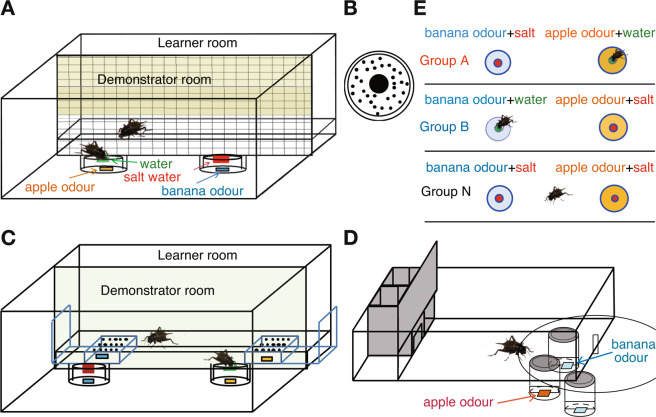
Experimental procedures. (**A**) Training arena. The training arena consists of a demonstrator room and a learner room, separated by a plastic net. Two drinking apparatuses containing water or 20% NaCl solution were placed in the demonstrator room. Filter papers soaked with apple essence and banana essence were placed under the apparatuses. A thirsty cricket (demonstrator) was placed in the demonstrator room and was allowed to freely visit the drinking apparatuses. Another thirsty cricket (learner) was placed in the learner room and allowed to observe the demonstrator room. (**B**) There were many small holes at the periphery of the drinking apparatus from which apple or banana odour diffused. (**C**) Training apparatus in which the use of olfactory cues for recognizing a demonstrator is prevented. Underneath the floor of the learner room in front of the drinking apparatus, a filter paper soaked with apple or banana essence (dissolved in aqueous ethanol) was placed so that the odour and water vapour can be emitted from small holes in the floor. (**D**) Test arena. There are two holes in the floor of the test arena that connect the floor with containers containing filter papers soaked with apple essence and banana essence dissolved in aqueous ethanol. The time to explore these odour sources was measured cumulatively. (**E**) In each experiment, crickets were divided into groups A, B and N according to the stimulus arrangements. In group A, the water-containing apparatus emitted apple odour and the saltwater-containing apparatus emitted banana odour, and the stimulus arrangement was reversed for group B. In group N, both of the apparatuses contained saltwater and each emitted either banana or apple odour.

Most of social learning trainings in this study were performed in a training arena, which consisted of a demonstrator room and a learner room separated by a plastic net (Fig. 1A). In the first experiment, a demonstrator cricket was placed in a demonstrator room and allowed to freely visit two drinking apparatuses (Fig. 1B) containing water or saltwater and emitting apple or banana odour, and a learner cricket was placed in a learner room and allowed to observe the behaviour of the demonstrator through a net for 8 min (For details of the procedures, see Materials and Methods). Crickets in three groups received training with different stimulus arrangements: In group A, the water-containing apparatus emitted apple odour and the saltwater-containing apparatus emitted banana odour, and the stimulus arrangement was reversed for group B (Fig. 1E). In group N, there were two apparatuses that both contained saltwater and emitted either apple or banana odour. As is described later, the demonstrator that visited water-containing apparatus touched the water-containing gauze net with the palpi or the mouth and then drunk it, whereas those that visited saltwater-containing apparatus left the apparatus soon after touching the saltwater-containing gauze net with the palpi or the mouth, and the learner that approached the plastic net in front of the apparatuses often touched the net with its antennae, palpi or mouth (Suppl. Movie 1).

**Figure 2:**
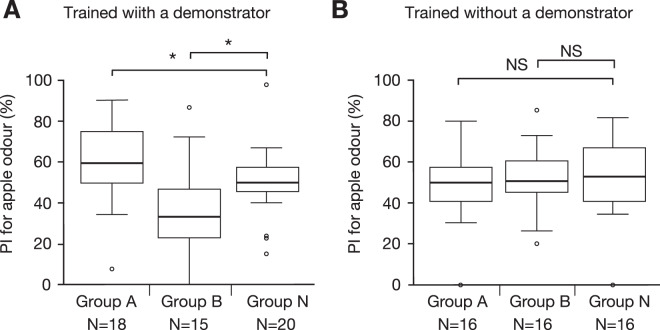
Odour preferences of learners in groups A, B and N trained with (**A**) and without (**B**) a demonstrator, tested at 24 hours after training. The preference indexes (PIs) for apple odour are shown as box plots. Results of statistical comparisons between the groups are shown (WCX test, *p < 0.05; NS, p > 0.05, with the P-value adjusted by Holm’s method).

Learners were tested for their relative preference between apple and banana odours 24 hours after training in an apparatus shown in Fig. 1D (for details, see Materials and Methods). The preference was measured by the preference index for apple odour, which is the ratio of the time spent visiting apple odour source relative to that spent visiting banana odour source during a 4 min test, shown as percentage. The preference for apple odour of learners in group A was significantly greater than that of learners in group N, and the preference for apple odour in group B was significantly less than that in group N (Fig. 2A, sample numbers shown in the figure and statistical results shown in Table 1). In a control experiment in which learners were subjected to training without a demonstrator (Fig. 2B), the preference for apple odour of learners did not significantly differ between groups A and N or between groups B and N, indicating that the presence of a conspecific is critical for learners to show different odour preferences according to different stimulus arrangements in training.

**Table 1:** Statistical results.

Groups	Test	W or U	P	Fig.
Living demonstrator: Groups A (n = 18) vs N (n = 20)	M-W	U = 248.5	0.047	2A
Groups B (n = 15) vs N	M-W	U = 82	0.048	2A
No demonstrator: Groups A (n = 16) vs N (n = 16)	M-W	U = 103	0.71	2B
Groups B (n = 16) vs N	M-W	U = 122.5	0.85	2B
Stay time of demonstrator: Group A (n = 19), apple vs banana	Welch t	T = 6.4	0.0000025	3A
Group B (n = 18), apple vs banana odours	Welch t	T = −6.0	0.0000069	3A
Group N (n = 20), apple vs banana odours	Welch t	T = 0.15	0.44	3A
Search time of learner: Group A (n = 19), apple vs banana odours	Welch t	T = 1.8	0.038	3B
Group B (n = 21), apple vs banana odours	Welch t	T = −3.1	0.0020	3B
Group N (n = 21), apple vs banana odours	Welch t	T = −0.49	0.31	3B
Search time of learner with a dead demonstrator: Group A (n = 21), apple vs banana odours	Welch t	T = 1.4	0.079	3C
Group B (n = 23), apple vs banana odours	Welch t	T = −0.80	0.21	3C
Group N (n = 22), apple vs banana odours	Welch t	T = 0.43	0.34	3C
Dead demonstrator: Groups A (n = 21) vs N (n = 22)	M-W	U = 111.5	0.0075	4A
Groups B (n = 23) vs N	M-W	U = 356	0.020	4A
Living demonstrator without visual cues: Groups A (n = 19) vs N (n = 15)	M-W	U = 234.5	0.0030	4B
Groups B (n = 13) vs N	M-W	U = 100	0.93	4B
Living demonstrator without olfactory cues: Groups A (n = 21) vs N (n = 21)	M-W	U = 223	0.019	4C
Groups B (n = 23) vs N	M-W	U = 77.5	0.0054	4C
Dead demonstrator without olfactory cues: Groups A (n = 28) vs N (n = 24)	M-W	U = 427	0.19	4D
Groups B (n = 29) vs N	M-W	U = 334	0.81	4D
Devalued after training with apple odour: Groups C (n = 21) vs D (n = 21)	M-W	U = 307.5	0.030	5
Devalued after training with banana odour: Groups C (n = 27) vs D (n = 28)	M-W	U = 254	0.038	5

M-W: Mann-Whitney test. Welch t: Welch’s t-test. P values were adjusted by Holm’s method in the cases of multiple comparisons.

**Figure 3:**
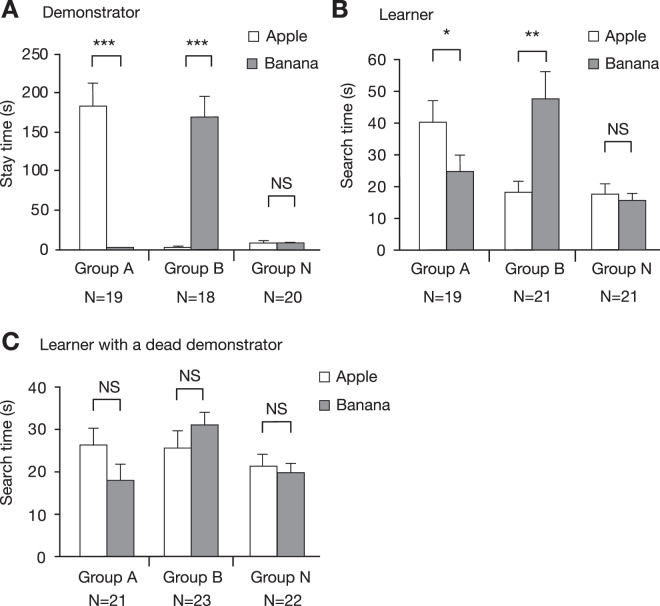
Stay time of demonstrators on water- and saltwater-containing apparatuses that emit apple or banana odours (**A**) and search time in which learners touched the net in front of the drinking apparatuses in groups A, B and N trained with a living (**B**) or a dead (**C**) demonstrator. The times spent visiting apparatuses with apple or banana odours are shown as mean and s.e.m. and were statistically compared in each group (Welch’s t-test, ***p < 0.001; **p < 0.01; *p < 0.05; NS, p > 0.05).

The behaviour of demonstrators and learners was quantitatively evaluated during training by the time that the demonstrator stayed on each of the apparatuses (Fig. 3A) and the time that the learners touched the net just in front of the apparatuses (Fig. 3B) that had been recorded on video. Demonstrators in groups A and B spent significantly more time on the water-containing apparatus than on the saltwater-containing apparatus (Fig. 3A). While the demonstrator remained in the water-contained apparatus, we observed that learners displayed a typical behavioural sequences in front of it, as shown in Suppl. Fig. S1. The learner typically (1) approached the net just in front of the apparatus where the demonstrator stayed, (2) touched the net with its antennae and then (3) touched the net with its palpi or mouth. Then the learner (4) stopped touching the net and (5) left the place or resumed touching the net (3). Hereafter, we refer to “search time” as the time that the learner spent touching the net with its antennae, palpi or mouth in front of the apparatus in a width range of 3 cm that matched the diameter of the drinking apparatus. The search time of learners in front of the water-containing apparatus, on which the demonstrator stayed for a long period of time, was significantly longer than that in front of the saltwater-containing apparatus, on which the demonstrator spent little time (Fig. 3B). Learners in group N did not spend much time touching the net in front of either of the apparatuses, with the search times being not significantly different in front of the two apparatuses. Thus, the learner exhibited preference for the odour of the apparatus on which a demonstrator stayed for a long period of time, which henceforth we refer to as “demonstrated” odour.

The simplest account of such increased preference for the odour demonstrated by a conspecific is that it is due to stimulus enhancement or sensitization^3^, i.e., attraction to a conspecific resulted in a longer exposure to an odour (Fig. 3B), which resulted in a higher preference for that odour. The results shown in Fig. 3B are in accordance with this explanation, but this possibility was refuted by results of the water devaluation experiment described in a later section.

### Social learning with a dead conspecific

**Figure 4:**
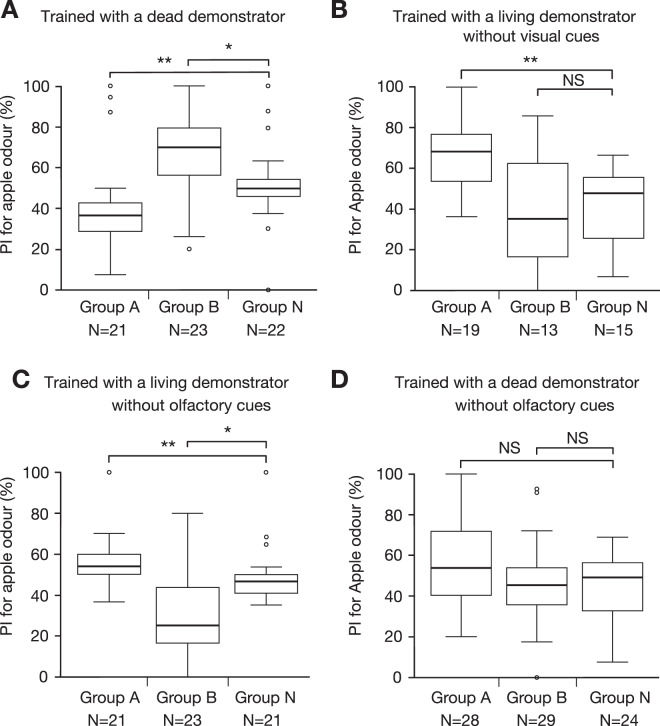
Odour preferences of learners in groups A, B and N trained with a dead demonstrator (**A**), those trained with a living demonstrator in the dark (**B**) and those trained with a living demonstrator (**C**) or a dead demonstrator (**D**) in a training apparatus that prevented the use of olfactory cues for recognizing the demonstrator. PIs for apple odour are shown as box plots. Results of statistical comparisons of odor preferences between the groups are shown (WCX test, **p < 0.01; *p < 0.05; NS, p > 0.05, with the P-values adjusted by Holm’s method).

We then investigated whether social learning can be achieved with a dead conspecific. We placed a freshly sacrificed conspecific on one of two drinking apparatuses that emitted apple (group A) or banana (group B) odour. In a control group (group N), a dead conspecific was placed in the floor equidistant from the two apparatuses. In the test performed 24 hours after training, apple odour preferences of learners in group A were significantly lower than those in group N, whereas those in group B were significantly higher than those in group N (Fig. 4A). Thus, the learner avoided the odour of the apparatus on which a dead demonstrator had been placed. We refer to this odour also as demonstrated odour.

The search times of learners at the net in front of the apparatuses with and without a dead demonstrator are shown in Fig. 3C. The search time of learners was slightly longer in the presence of a dead demonstrator than in the absence of a dead demonstrator in both groups A and B, but the difference was not statistically significant. Learners in group N exhibited no significant difference in the search times at the net in front of the two apparatuses. Thus, aversion to the demonstrated odour (Fig. 4A) is probably not due to longer exposure to that odour (or habituation).

### Effects of social learning training in the dark

Next, we attempted to clarify which sensory cues are required for recognition of a living or dead conspecific to achieve appetitive or aversive social learning. We first wanted to know whether learning with a living demonstrator can be achieved in the dark, i.e., without using visual cues for recognizing a conspecific. In the test performed 24 hours after training, apple odour preferences of learners in group A were significantly greater than those in group N, whereas those in group B did not significantly differ from those in group N (Fig. 4B). Thus, social learning with a living conspecific can be achieved without using visual cues for recognizing a conspecific, at least in one of the two stimulus conditions.

We also performed social learning training with a dead demonstrator in the dark, but we failed to obtain meaningful results since the majority of the learners visited the odour sources for less than 10 sec. In such cases, we routinely excluded the data from analysis considering that the crickets were less motivated to visit odour sources. More efforts are needed to establish procedures to allow investigation of social learning with a dead demonstrator in the dark.

### Effects of deprivation of olfactory cues for recognizing a conspecific

Next, we investigated whether crickets can achieve social learning with a living or dead conspecific when they were deprived of using olfactory cues for recognizing a conspecific. In these experiments, we used a new training apparatus (Fig. 1C) in which the learner room was separated from the demonstrator room by a transparent Lucite plate, so that the learner could see the demonstrator but not smell it. Instead, a filter paper soaked with apple or banana essence, diluted in aqueous ethanol, was placed underneath the floor of the learner room in front of the drinking apparatuses, so that the odour and water vapour could diffuse into the learner’s room. After training with a living demonstrator (Fig. 4C), apple odour preferences of learners in group A were significantly greater than those in group N, whereas those in group B were significantly less than those in group N, indicating that social learning with a living demonstrator can be achieved without using olfactory cues for recognizing a demonstrator.

In the social learning experiment with a dead demonstrator, apple odour preferences of leaners did not significantly differ between groups A and N or between groups B and N (Fig. 4D), suggesting that it is very difficult to achieve aversive social learning with a dead conspecific without using olfactory cues for recognizing it. It seems that crickets visually discriminate living conspecific and motionless one and learn from the former (Fig. 4C) but not from the latter (Fig. 4D) when olfactory cues are not available.

### Effects of devaluation of water before the post-training test

**Figure 5:**
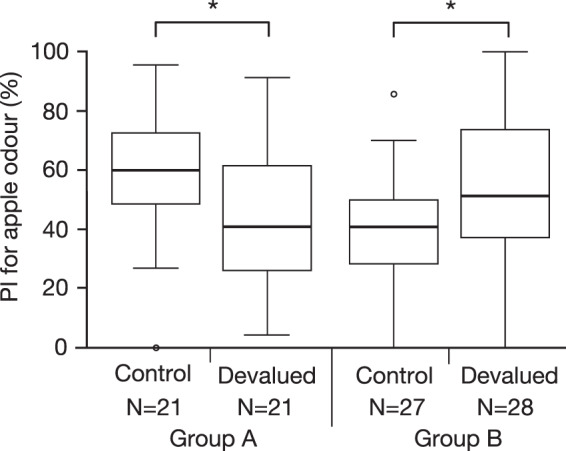
Effects of devaluation of water on odour preferences of learners in the post-training test. Learners in groups A and B were trained with a living conspecific. The next day, learners in each group were further divided into a subgroup in which learners were given water until satiation (devalued) and a subgroup in which no water was given (control). Thirty minutes later, the learners received test, and PIs for apple odour are shown as box plots. Results of statistical comparisons of odor preferences between the control and devalued groups are shown (WCX test, *p < 0.05).

Finally, we investigated the effects of providing water until satiation and hence reducing the value of water before post-training test on responses to learned odour. We used such a devaluation procedure for examining if responses to a CS after pairing of it with water US were governed by the association between the CS and the US^26^. Learners in groups A and B were subjected to training with a living conspecific and then they were further divided into two groups. Learners in one group (water-devalued learners) were given water until satiation, whereas those in the other group (control learners) were not given water. Preferences for the demonstrated odour of water-devalued learners were significantly less than those of control learners in groups A and B (Fig. 5). The results suggest that crickets are motivated to find water when visiting learned odour in the post-training test.

## Discussion

### Appetitive social learning with a living conspecific

Social learning plays critical roles for survival of animals in the natural environment and it is found in many groups of animals including insects^3,4^, but there are few experimental paradigms that allow detailed analysis of associative processes that enables social learning^4^, which hampers the progress of our understanding of neural mechanisms of social learning. In this study, we established procedures for social learning by modifying those for Pavlovian conditioning in the field cricket *Gryllus bimaculatus*, in which we have investigated neural mechanisms of Pavlovian conditioning in some detail^18–23,27,28^. We observed that the crickets exhibited preference for the odour of a drinking apparatus on which a living demonstrator stayed for a long period of time over the odour of an apparatus on which the demonstrator spent little time (Figs. 2A and 3A). We also found that crickets avoided the odour of an apparatus on which a dead conspecific had been placed (Fig. 4A). There has been a report on social learning in another species of crickets, the wood cricket *Nemobius sylvestris*. In this species, the predator-avoidance behaviour of learner crickets was changed after observing the behaviour of demonstrator crickets that had experienced a predator^29^. In that study, a 6-hour period of observation was required to achieve 24-hour memory. In the present study, in contrast, only an 8-min observation period was sufficient for achieving 24-hour memory, the time frame of which matches protein synthesis-dependent long-term memory in crickets^30^. Importantly, the experimental paradigms described here allow analysis of associative processes underlying social learning in well-controlled stimulus arrangements, which has been achieved only very rarely in any social learning systems of animals. Finally, the excellent social learning capability of crickets, which are subsocial insects, confirms a previous suggestion^29^ that social learning capabilities are not confined to highly social animals but are ubiquitous among animals with diverse lifestyles.

### Sensory cues used to recognize a living or dead conspecific for social learning

We found that social learning with a living demonstrator was achieved when learners were deprived of using visual (Fig. 4B) or olfactory cues (Fig. 4C) for recognizing a demonstrator, although social learning without visual cues was achieved in only one of the two stimulus conditions. It is most likely that olfactory cues were used for recognition of a conspecific in the case of social learning without visual cues, possibly by cuticular hydrocarbons, which are known to be used for recognition of conspecifics in insects^31^. However, the possible contribution of auditory cues that might have been produced by subtle movement of a conspecific cannot be ruled out, although we found no evidence of such noise production from sound recording. In addition, there were cases (<10%) in which the demonstrator exhibited stridulation during training, and sounds might have served as additional cues for recognition of a conspecific in such cases.

We observed no social learning with a dead conspecific when the use of olfactory cues for recognizing a dead demonstrator was prevented, suggesting that odours of a dead conspecific are needed to achieve aversive social learning (Fig. 4D). In house crickets, *Acheta domesticus*, it has been reported that oleic acid serves as a cue for recognition of dead conspecifics^32^ and that crickets exhibited aversion to an odour after presenting it in compound with oleic acid for 24 hours^33^. Whether oleic acid or other chemicals are used for recognition of a dead conspecific in *Gryllus bimaculatus* remains to be studied.

### Possible associative processes underlying social learning with a living or dead conspecific

An important question to be addressed is by what mechanisms the social learning described here is achieved. The simplest account of social learning with a living conspecific is that it is due to stimulus enhancement or sensitization, i.e., the learners were attracted to the conspecific and this led to a long exposure to an odour and, as a consequence, to enhanced responses to that odour^3^. The second-simplest account may be that an association is formed between the odour and the living conspecific during training and that this association governs response to the demonstrated odour in the post-training test. The results of our water devaluation experiment (Fig. 5), however, were not consistent with these accounts as is discussed below.

Crickets that had received social learning training with a living conspecific and then given water until satiation exhibited no preference for the demonstrated odour (Fig. 5). We thus reasoned that the learners were motivated to search for water when responding to the demonstrated odour, which suggests that an association between the odour and water is formed during social learning training and it is this association that governs responses to the demonstrated odour. This is analogous to our previous finding that crickets that had received Pavlovian training to associate an odour (CS) with water (US) and then given water until satiation exhibited no preference for the conditioned odour in a subsequent test^23^. A question that arises is by what mechanisms such an odour-water association is formed by social learning training. One possibility is that it is formed by second-order conditioning, an associative learning process proposed to account for social learning in bumblebees, in which bees learn to use the colour of the flower to find a sucrose solution when they experienced conspecifics staying on the flower of that colour^5,34^. Following this second-order conditioning account of social learning, animals associated a conspecific CS1 with an appetitive US (sucrose solution in the case of bees and water in the case of crickets) and then they also associated another stimulus CS2 (color in the case of bees and odour in the case of crickets) with the conspecific (CS1). This results in the subject associating CS2 and US (the colour and sucrose solution in the case of bees and the odour and water in the case of crickets). We have reported that crickets exhibit second-order conditioning with odour as CS1, visual pattern as CS2 and water as US^22^. If this account is correct, crickets that had been reared in isolation and had no prior experience observing conspecifics drinking water do not achieve social learning with a living conspecific. This prediction needs to be tested.

Avoidance of the odour demonstrated by a dead conspecific seems not be due to habituation or a long exposure to the demonstrated odour since the time that the learners spent exploring the net in front of an apparatus with a dead conspecific was not significantly longer than that in front of the other apparatus (Fig. 3C). The second simplest account of aversive social learning with a dead conspecific is that a dead conspecific served as an aversive US. Based on this assumption, an association between the odour CS and the dead conspecific US was formed during training and this association is what governs aversion to the demonstrated odour. If this is the case, devaluation of water before the post-training test should have no effect on aversive responses to the demonstrated odour. This possibility needs to be tested.

### Future perspectives: toward elucidation of neural mechanisms underlying social learning

One of our future subjects is to examine the roles of octopamine and dopamine neurons for achieving appetitive and aversive forms of social learning in crickets, since our pharmacological, RNAi, and genome editing studies all suggested that octopamine and dopamine neurons convey signals about appetitive and aversive US for achieving Pavlovian conditioning with water and saltwater US, respectively^18–21^. Therefore, if aversive social learning with a dead conspecific is achieved by Pavlovian conditioning, we can deduce that dopamine neurons mediate signals about a dead conspecific for achieving learning and hence administration of dopamine receptor antagonist before training impairs learning. Moreover, we have shown that activation of octopamine neurons is required for achieving appetitive second-order conditioning in crickets^22^. Assuming that this finding is applicable to social learning with a living conspecific, we can deduce that octopamine neurons that mediate signals about water are activated when the learner observes a living conspecific and that this activation enhances the efficacy of synapses that form the association between the odour CS2 and water US^22^.

In conclusion, we suggest that a living conspecific is recognized by either visual or olfactory cues for appetitive social learning, whereas olfactory cues are used to recognize a dead conspecific for aversive social learning in crickets. We also suggest and that social learning with living or dead conspecific can be accounted for by second-order or first-order Pavlovian conditioning, respectively. We have reported that octopamine neurons play critical roles for achieving appetitive second-order conditioning^22^, whereas dopamine neurons play critical roles for achieving aversive first-order conditioning^18,20,21^ in crickets, and the experimental paradigms developed here will pave the way for detailed research on the roles of octopamine and dopamine neurons for achieving appetitive and aversive forms of social learning.

## Materials and Methods

### Animals

Adult make crickets, *Gryllus bimaculatus*, at 1 week after the imaginal molt were used. They were reared in a 12 h:12 h light:dark cycle at 27 ± 2 °C and were fed a diet of insect pellets and water ad libitum. Three days before the start of the experiment, about 50 animals were individually placed in 100-ml glass beakers and deprived of drinking water to enhance their motivation to search for water.

### Training arena

A cricket (learner) placed in a learning room was allowed to observe the behaviour of another cricket (demonstrator) in a demonstrator room through a plastic net (3.9 × 3.9 mm; thickness, 0.7 mm) (Fig. 1a). The training arena (19.5 × 10.5 × 8.5 cm) was made of white Lucite plates, and paper towels were fixed on the floor of the arena by glue. The upper part of the net was covered with plastic tapes to prevent the cricket from climbing. Two drinking apparatuses (3 cm in diameter and 1.4 cm in height, Fig. 1b) were placed in the demonstrator room. At the centre of the drinking apparatus, there was a hole in which a gauze net soaked with water or 20% saltwater (NaCl solution) was placed. A small piece of filter paper soaked with apple or banana essence, dissolved in aqueous ethanol, was placed underneath the apparatus. The periphery of the apparatus had many small holes so that odour diffusion was ensured. The floor of the learner room was 1.5 cm higher than that of the demonstrator room, helping the learner to observe the demonstrator’s behaviour on the drinking apparatus.

### Training procedure

In experiments with a living conspecific, an adult male cricket placed in a beaker was gently released in the demonstrator room and then a learner was similarly released in the learner room for 8 min. At 4 min from the start of training, the locations of the two apparatuses were exchanged using tweezers. The behaviour of the demonstrator and the learner was recorded by a video camera. Data were discarded when there was direct contact between the crickets with their antennae, palpi, mouths or other parts of the body. In experiments with a dead conspecific, a dead adult male cricket 1 week after imaginal molt was used as a demonstrator. Dead demonstrators were sacrificed by chilling for 2–24 hours and were then returned to room temperature (25–28°) for 30 min before the experiment.

In an experiment to clarify whether this type of social learning relies on visual cues, training with a living or dead demonstrator was performed in the dark, and the behaviour of the demonstrator and the learner was monitored by an infrared CCD camera, with the peak wavelength of the infrared light being 940 nm. Dim red light from LED arrays, for which photoreceptors of the compound eyes of *Gryllus bimaculatus* are not sensitive^24^, was used for setting up the experiment. In another series of experiments, we investigated whether social learning is possible in the absence of olfactory cues for recognizing a conspecific by using a new training arena in which the learner room was partitioned from the demonstrator room by a transparent Lucite plate (2 mm in thickness) (Fig. 1C). Under the floor of the learner room in front of the drinking apparatus of the demonstrator room, saucers containing filter papers soaked with either banana or apple essence, dissolved in aqueous ethanol, were placed. There were many small holes in the floor above the saucers so that the odour and water vapour can diffuse into the learner room. As such, the training was designed so that a learner can sense apple or banana odour and water vapor in front of the drinking apparatus containing water or saltwater and so that the learner can use visual cues but not olfactory cues for recognizing the demonstrator. This training was performed under bright light (white fluorescent lamp, 4800 lux) since in our preliminary experiment this learning was less well achieved under standard room light (680 lux). However, it remains to be clarified if such bright light is needed for achieving this learning.

For evaluation of the behaviour of the demonstrator and the learner during training, the time that the demonstrator stayed at each of the two drinking apparatuses and the time that the learner explored the net just in front of each apparatus were recorded by video. For definition of the latter, see Results section. Welch’s t-test was used for statistical comparison of the search times for the two apparatuses.

### Devaluation of water before the post-training test

In the last experiment, we tested the effect of reward devaluation procedure on learner’s responses to learnt odour^26^. In this experiment, crickets received social learning training and 24 hours later they were given water until satiation by a pipette until they stopped drinking. Thirty minutes later, they received an odour preference test. We have shown that crickets that received Pavlovian conditioning training to associate an odour CS with water US and then received such a devaluation procedure before the test did not exhibit response to conditioned odor and hence we concluded that the association between the odour and water governs responses to the odour^23^. In that study we also showed that providing water until satiation does not alter innate odour preferences of untrained crickets and that it does not reduce the sensory or motor functions or motivation necessary to respond to the conditioned odour^23^.

### Odour preference test

For evaluation of the effect of social learning, the learner’s relative preference between apple and banana odours was tested 24 hours after training. The test was performed in a test arena (Fig. 1d)^17^ in which there were two holes in the floor connecting the floor with two containers that contained filter papers soaked with apple or banana essence dissolved in aqueous ethanol. The upper part of the container was covered with a gauze net. In the test, a cricket was released in the test arena and allowed to freely visit the odour sources for 4 minutes. The locations of the odours were exchanged 2 minutes after the start of the test by rotating the container holder. The time that the cricket spent touching the gauze net covering each container with its mouth or palpi was measured cumulatively.

### Statistical evaluation

The relative odour preference was measured by the preference index (PI) for apple odour, which is the ratio of the search time of apple odour relative to that of banana odour, shown as percentage. If the total time to visit the odour sources was less than 10 sec, we considered that the cricket was less motivated, and the data were discarded. Such crickets were less than 10% of the tested crickets in all experiments except in the experiment in which social learning was examined with a dead conspecific in the dark (see Results section). Wilcoxon’s test was used to compare odour preferences between different groups^18,19^. Holm’s method was used to adjust the p-value in the case of multiple comparisons. P < 0.05 was considered statistically significant.

## Supplementary information

Supplementary Information.

Supplementary Information 2.

## Data Availability

The behavioral datasets used in the current study and information about detailed experimental procedures are available from the corresponding author on reasonable request.

## References

[1] Fiorito G, Scotto P (1992). Observational learning in Octopus. vulgaris. Science.

[2] Leadbeater. E, Chittka L (2009). Bumble-bees learn the value of social cues through experience. Biol. Lett..

[3] Zentall TR (2011). Perspectives on observational learning in animals. J. Comp. Psychol..

[4] Heyes C (2012). What’s social about social learning?. J. Comp. Psychol..

[5] Dawson EH, Avargue’s-Weber A, Chittka L, Leadbeater E (2013). Learning by observation emerges from simple associations in an insect model. Curr. Biol..

[6] Dunlap, A. S., Nielsen, M. E., Dornhaus, A. & Papaj, D. R. Foraging bumble bees weigh the reliability of personal and social information. *Curr Biol.* **26**, 1195–1199 (2016). 10.1016/j.cub.2016.03.009 27133871

[7] Dawson EH, Chittka L (2014). Bumblebees (*Bombus terrestris*) use social information as an indicator of safety in dangerous environments. Proc. R. Soc. B..

[8] Mery F (2009). Public versus personal information for mate copying in an invertebrate. Curr. Biol..

[9] Joiner J, Piva M, Turrin C, Chang SWC (2017). Social learning through prediction error in the brain. Sci. Learn..

[10] Monier M, Nöbel S, Danchin E, Isabel G (2019). Dopamine and serotonin are both required for mate-copying in *Drosophila melanogaster*. Front. Behav. Neurosci..

[11] Carcea I, Froemke RC (2019). Biological mechanisms for observational learning. Curr. Opin. Neurobiol..

[12] Yao M (2009). The ancient chemistry of avoiding risks of predation and disease. Evol. Biol..

[13] Iglesias TL, Stetkevich RC, Patricelli GL (2014). Dead heterospecifics as cues of risk in the environment: Does size affect response?. Behaviour.

[14] Grüter C, Leadbeater E (2014). Insights from insects about adaptive social information use. Trends Ecol. Evol..

[15] Hedwig B (2006). Pulses, patterns and paths: neurobiology of acoustic behaviour in crickets. J. Comp. Physiol. A.

[16] Matsumoto Y, Mizunami M (2000). Olfactory learning in the cricket *Gryllus bimaculatus*. J. Exp. Biol..

[17] Matsumoto Y, Mizunami M (2002). Temporal determinants of long-term retention of olfactory memory in the cricket *Gryllus bimaculatus*. J. Exp. Biol..

[18] Unoki S, Matsumoto Y, Mizunami M (2005). Participation of octopaminergic reward system and dopaminergic punishment system in insect olfactory learning revealed by pharmacological study. Eur. J. Neurosci..

[19] Unoki S, Matsumoto Y, Mizunami M (2006). Roles of octopaminergic and dopaminergic neurons in mediating reward and punishment signals in insect visual learning. Eur. J. Neurosci..

[20] Awata H (2015). Knockout crickets for the study of learning and memory: Dopamine receptor Dop1 mediates aversive but not appetitive reinforcement in crickets. Sci. Rep..

[21] Awata H (2016). Roles of OA1 octopamine receptor and Dop1 dopamine receptor in mediating appetitive and aversive reinforcement revealed by RNAi studies. Sci. Rep..

[22] Mizunami M (2009). Roles of octopaminergic and dopaminergic neurons in appetitive and aversive memory recall in an insect. BMC Biol..

[23] Mizunami M (2019). Development of behavioural automaticity by extended Pavlovian training in an insect. Proc. R. Soc. B..

[24] Doi N, Toh Y (1992). Modification of cockroach behavior to environmental humidity change by dehydration (Dictyoptera: Blattidae). J. Insect Behav..

[25] Zufall F, Schmitt M, Menzel R (1989). Spectral and polarized light sensitivity of photoreceptors in the compound eye of the cricket (*Gryllus bimaculatus*). J. Comp. Physiol. A..

[26] Holland PC, Rescorla RA (1975). The effect of two ways of devaluing the unconditioned stimulus after first- and second-order appetitive conditioning. J. Exp. Psychol. Anim. Behav. Process..

[27] Terao K, Matsumoto Y, Mizunami M (2015). Critical evidence for the prediction error theory in associative learning. Sci. Rep..

[28] Terao K, Mizunami M (2017). Roles of dopamine neurons in mediating the prediction error in aversive learning in insects. Sci. Rep..

[29] Coolen I, Dangles O, Casas J (2005). Social learning in noncolonial insects?. Curr. Biol..

[30] Matsumoto Y, Noji S, Mizunami M (2003). Time course of protein synthesis-dependent phase of olfactory memory in the cricket *Gryllus bimaculatus*. Zool. Sci..

[31] Howard RW, Blomquist GJ (2005). Ecological, behavioral, and biochemical aspects of insect hydrocarbons. Annu. Rev. Entomol..

[32] Aksenov V, Rollo DC (2017). Necromone death cues and risk avoidance by the cricket *Acheta domesticus*: Effects of sex and duration of exposure. J. Insect Behav..

[33] Shephard AM, Aksenov V, Rollo DC (2018). Conspecific mortality cues mediate associative learning in crickets. Acheta domesticus (Orthoptera: Gryllidae) J. Orthoptera Res..

[34] Giurfa M (2012). Social learning in insects: a higher-order capacity?. Front. Beahav. Neuroci..

